# Serum uric acid level is associated with glomerular ischemic lesions in patients with primary membranous nephropathy: an analytical, cross-sectional study

**DOI:** 10.1038/s41598-024-57813-5

**Published:** 2024-03-29

**Authors:** Yamin Yu, Juan Zheng, Jie Li, Xiuzhen Li, Zewei Liu, Ruiheng Yang, Hong Hong, Junjun Zhang

**Affiliations:** 1 Department of Nephrology, The First Affiliated Hospital of Zhengzhou University, Zhengzhou, People’s Republic of China; 2 Department of Joint Laboratory for Translational Medicine Research, Liaocheng People’s Hospital, Liaocheng, People’s Republic of China; 3 Department of Nephrology, Liaocheng People’s Hospital, Liaocheng, People’s Republic of China

**Keywords:** Nephrology, Risk factors

## Abstract

To investigate the relationship between serum uric acid level and glomerular ischemic lesions (GIL) in patients with primary membranous nephropathy (PMN) and identify relevant risk factors. A total of 201 patients with PMN but normal renal function confirmed by renal biopsy executed in the Liaocheng People’s Hospital, China, during January 2020-January 2023 were analyzed retrospectively. The enrolled patients were divided into a hyperuricemia group and a normal serum uric acid group (control group) according to their serum uric acid levels. Then, the participants were further divided into a non-GIL group or a GIL group based on the patient’s renal biopsy results. The two groups’ clinical and pathological data and meaningful indicators for differences were analyzed by binary logistic regression analysis. Additionally, the serum uric acid level prediction value on GIL was investigated using receiver operating characteristic (ROC) curves. Compared with the control group, the hyperuricemia group exhibited high serum uric acid, the prevalence of GIL, serum albumin, the prevalence of hypertension, and low-density lipoprotein cholesterol (LDL) levels (*P* < 0.05). Compared with the non-GIL group, the GIL group exhibited were older, had enhanced serum uric acid, serum albumin, and an increased prevalence of tubular atrophy/interstitial fibrosis (TA/IF), arteriolosclerosis, and low eGFR levels (*P* < 0.05). The binary logistic regression analysis revealed that the serum uric acid and the TA/IF are independent risk factors of GIL (*P* < 0.05). The AUC of ROC of GIL of PMN patients, predicted based on the serum uric acid concentration, was 0.736 (*P* < 0.05), wherein the threshold = 426.5 μmol/L and the Youden’s index = 0.41. Serum uric acid concentration and the TA/IF are independent risk factors of GIL in patients with PMN, and the former exhibits prediction value on GIL in patients with PMN.

## Introduction

Membranous nephropathy (MN) is a group of diseases characterized by the deposition of immune complexes under epithelial cells on the outer side of the glomerular basement membrane (GBM), with diffuse thickening of GBM^1^. MN can be divided into primary and secondary forms, and those with unknown causes are referred to as primary membranous nephropathy (PMN). PMN is a common primary nephrotic syndrome observed in the middle-aged and elderly. Recently, there has been a significant increase in MN cases in younger patients in China, with approximately 30% of these progressing to end-stage renal disease after several years^2,3^. Renal ischemia plays an essential role in the development of chronic renal disease. Specifically, research has shown that long-term hypoxia–ischemia leads to atrophy of the nephron, decline of glomerular filtration function, and aggravation of renal injury^4^. It is widely known that hyperuricemia is closely related to cardiovascular and cerebrovascular diseases^5,6^. However, whether hyperuricemia aggravates ischemic renal disease remains unknown. The previous studies have demonstrated a significantly higher incidence of glomerular ischemic lesions in IgA nephropathy patients with hyperuricemia compared to those with normal uric acid levels, and there is an independent correlation between the elevation of serum uric acid levels and the development of glomerular ischemic disease. These findings suggest that hyperuricemia may contribute to the occurrence of glomerular ischemic lesions in IgA nephropathy^7^. However, the impact of uric acid levels on the occurrence of ischemic lesions in primary membranous nephropathy has been scarcely investigated. Since hyperuricemia is usually secondary to chronic renal insufficiency, only patients with typical glomerular filtration rates were assessed in the current study to avoid confounding factors. This study aimed to assess the clinical and pathological data of PMN patients to investigate the relationship between the level of serum uric acid and glomerular ischemic lesions (GIL) in patients with PMN with normal renal function.

## Subjects and method

### Subjects and inclusion criteria

From January 2020 to January 2023, 265 cases of MN who received renal biopsies in Liaocheng People’s Hospital were assessed. The inclusion criteria were: (a) a renal biopsy diagnosis confirming typical PMN; (b) glomerular filtration rate (eGFR) > 90ml/(min.1.73m^2^) at the time of renal biopsy; and (c) availability of complete clinical, pathological, and laboratory data. The exclusion criteria were as follows: (a) individuals with a glomerulus count of less than 10 in renal tissues under light microscopy in the selected samples; (b) biopsy-confirmed cases of atypical MN; (c) those with secondary MN, such as MN caused by infections (e.g., hepatitis B virus and hepatitis C virus), malignancies (e.g., solid tumors and multiple myeloma), drugs (non-steroidal anti-inflammatory drugs), or autoimmune diseases (systemic lupus erythematosus); (d) patients who had taken medications affecting uric acid metabolism within the past month: uric acid-lowering drugs including febuxostat, allopurinol, and benzbromarone; diuretics including furosemide, bumetanide, hydrochlorothiazide, and torasemide; immunosuppressants including cyclosporine, tacrolimus, glucocorticoids; quinolone antibiotics including ofloxacin and moxifloxacin; acarbose; SGLT-2 inhibitors such as empagliflozin and canagliflozin; fenofibrate; atorvastatin; losartan; (e) individuals with an eGFR less than 90 mL/(min.1.73m2); (f) individuals with MN combined with other pathological types; (g) along with patients having incomplete clinicopathological data. Based on these exclusion criteria, 64 patients were excluded, resulting in 201 cases of PMN being assessed. The study protocol was approved by the Ethics Committee of Liaocheng People’s Hospital. All methods were carried out in accordance with relevant guidelines and regulations (e.g. Helsinki guidelines).

## Methods

### Clinical data

Clinical data and laboratory indicators were collected from patients during a renal biopsy, including age, sex, serum creatinine (Scr), glomerular filtration rate (eGFR), serum uric acid level (UA), hemoglobin (Hb), red blood cell (RBC) count, white blood cell (WBC) count, platelet (PLT) count, total cholesterol (TC), triglyceride (TG), low-density lipoprotein (LDL), serum albumin (Alb), urinary RBC, and 24 h urine protein quantitation, serum anti-PLA2R antibody (anti-PLA2R).

### Grouping

Next, the participants were divided into the normal serum uric acid group (control group) and the hyperuricemia group according to the serum uric acid level. The diagnostic criteria of hyperuricemia was > 420 μmol/L for male, and > 357 μmol/L for females. Next, the renal biopsy results divided the patients into the non-glomerular ischemic lesion (GIL) or GIL groups. The evaluation criteria for GIL encompassed the identification of glomerular ischemic sclerosis, ischemic atrophy, or ischemic shrinkage through renal biopsy analysis.

### Statistical analysis

Statistical analysis was conducted using IBM SPSS Statistics for Windows, version 27.0 (IBMCorp., Armonk, N.Y., USA, www.ibm.com/spss). All continuous data were assessed for normality using the Kolmogorov–Smirnov test. Normally distributed data between the two groups were compared by t-test and expressed as mean ± SD ($\overline{x }$ ± s). Non-normally distributed data were compared using the Mann–Whitney U test, and the data were expressed as a median and interquartile range [M (Q1, Q3)]. Categorical variables were expressed as frequency or percentage and were compared using the χ^2^ test. Binary Logistic regression analysis investigated the independent risk factors contributing to GIL. Receiver operating characteristic (ROC) curves were used to investigate the sensitivity and specificity of PMN patients’ GIL serum uric acid levels. A *P*-value of < 0.05 was used to indicate statistical significance.

### Ethics approval and consent to participate

This study was approved by the Ethics Committee of the Liaocheng People’s Hospital. Since this was a retrospective study and all data was anonymous, informed consent was waived by the Ethics Committee of Liaocheng People's Hospital.

## Results

### Comparison of clinical data between the control and the hyperuricemia groups

There were 146 cases in the control group, constituting 89 males and 57 females, aged an average of 48.97 ± 11.57 years (14–73 years). The average serum uric acid level was 326.57 ± 59.19 μmol/L. There were 55 cases in the hyperuricemia group, of which 37 were males, and 18 were females, and the age ranged from 26 to 71, averaging 50.09 ± 9.79 years. The serum uric acid level was 459.16 ± 41.15 μmol/L. In this study, the prevalence of hyperuricemia was 27.4% (55/201), and the sex, age, hemoglobin, serum creatinine, eGFR, proteinuria (g/24 h), total cholesterol, and triglyceride of the two groups were similar. In contrast, the serum uric acid level, the prevalence of GIL, serum albumin, the prevalence of hypertension, and LDL of the hyperuricemia group were significantly higher than those of the control group (*P* < 0.05) (Table 1).

**Table 1:** Comparison of clinical data between the control group and the hyperuricemia group [n (%), $\overline{x }$ ± s, M (Q1, Q3)].

Clinical factor	Control Group (n = 146)	Hyperuricemia Group (n = 55)	*t*/χ^2^/Z	*P*-value
Sex				
M	89 (61.0)	37 (67.3)	0.681	0.409
F	57 (39.0)	18 (32.7)		
Age (years old)	48.97 ± 11.57	50.09 ± 9.79	− 0.636	0.526
Serum uric acid level (μmol/L)	326.57 ± 59.19	459.16 ± 41.15	− 17.92	< 0.001
Glomerular ischemic lesion				
Yes	38 (26.0)	38 (69.1)	31.51	< 0.001
No	108 (74.0)	17 (30.9)		
Hemoglobin (g/L)	135.70 ± 15.95	136.08 ± 15.66	− 0.150	0.081
Serum albumin (g/L)	23.71 ± 5.48	26.49 ± 5.40	− 3.215	0.002
Serum creatinine (μmol/L)	65.62 ± 13.87	67.82 ± 12.91	− 1.021	0.308
eGFR [ mL/(min.1.73m^2^)]	107.32 ± 10.49	105.20 ± 9.36	1.309	0.192
Proteinuria (g/24 h)	5.437 (3.465, 7.954)	5.092 (3.125, 7.543)	− 0.937	0.349
Hypertension				
Yes	59 (40.7)	33 (60.0)	5.986	0.014
No	86 (59.3)	22 (40.0)		
TC (mmol/L)	6.93 (5.65, 8.67)	7.76 (6.22, 9.40)	− 1.844	0.065
TG (mmol/L)	1.95 (1.49, 2.62)	2.1 (1.43, 2.97)	− 0.976	0.329
LDL (mmol/L)	4.57 (3.51, 5.76)	5.29 (3.72, 6.78)	− 2.262	0.024

### Comparison of pathological data of the GIL group and the non-GIL group

There were 125 cases in the non-GIL group, consisting of 73 males and 52 females. The age range varied from 14 to 70 years, averaging 47.74 ± 11.39 years. Conversely, the GIL group included 76 cases comprising 53 males and 23 females. The age range in this group spanned 24 to 73 years, and the average age was 51.80 ± 10.19 years. In this study, the prevalence of GIL was 37.8% (76/201). Anti-PLA2R antibodies were detected in 70.15% of the patients included in this study. Herein, sex, hemoglobin, white blood cell count, platelet count, serum creatinine, TC, TG, LDL, urinary RBC, proteinuria (g/24 h), anti-PLA2R, the prevalence of hypertension, prevalence of glomerular sclerosis, prevalence of segmental glomerulosclerosis, and prevalence of arteriolar hyalinosis were similar between the two groups. However, in the GIL group age, serum uric acid level, serum albumin, the prevalence of renal tubular atrophy/interstitial fibrosis, and prevalence of arteriolosclerosis was significantly higher in the GIL group than the non-GIL group (*P* < 0.05). Additionally, the eGFR was significantly lower in the GIL group than in the non-GIL group (*P* < 0.05) (Table 2).

**Table 2:** Clinical and pathological data of the GIL group and the non-GIL group [n (%), $\overline{x }$ ± s, M (Q1, Q3)].

Item	GIL Group (n = 76)	Non-GIL Group (n = 125)	*t*/χ^2^/Z value	*P-*value
Sex				
M	53 (69.7)	73 (58.4)	2.597	0.107
F	23 (30.3)	52 (41.6)		
Age	51.80 ± 10.19	47.74 ± 11.39	2.548	0.012
Hypertension				
Yes	41 (53.9)	51 (40.8)	3.291	0.07
No	35 (46.1)	74 (59.2)		
Serum uric acid level (μmol/L)	403.69 ± 79.64	338.02 ± 70.84	6.007	< 0.001
Serum creatinine (μmol/L)	67.04 ± 13.03	64.36 ± 13.13	1.408	0.161
eGFR [mL/(min.1.73m^2^)]	104.97 ± 8.83	108.83 ± 10.76	− 2.636	0.009
Hemoglobin (g/L)	135.02 ± 15.28	136.29 ± 16.21	− 0.550	0.583
WBC (× 10^9^/L)	6.37 (5.325, 7.975)	6.34 (5.04, 7.29)	− 0.746	0.46
PLT (× 10^9^/L)	242 (211, 290)	251 (209, 300)	− 0.018	0.986
Serum albumin (g/L)	25.67 ± 5.39	23.75 ± 5.60	2.362	0.019
Proteinuria (g/24 h)	5.246 (3.094, 7.534)	5.384 (3.501, 8.147)	-0.633	0.527
Blood urine (/μL)	27.92 (16.85, 65.225)	35.6 (19.2, 74.4)	− 1.2	0.23
TC (mmol/L)	7.24 (6.045, 8.475)	7.17 (5.62, 9.23)	− 0.345	0.73
TG (mmol/L)	2.03 (1.485, 2.77)	1.95 (1.44, 2.62)	− 0.784	0.433
LDL (mmol/L)	4.97 (3.915, 6.095)	4.63 (3.45, 5.98)	− 0.577	0.564
anti-PLA2R(RU/ml)	38.09(17.95,74.14)	31.47(5.33,97.48)	− 1.043	0.297
Glomerular sclerosis				
No	51 (67.1)	93 (74.4)	1.238	0.266
Yes	25 (32.9)	32 (25.6)		
Glomerular segmental sclerosis				
No	69 (90.8)	107 (85.6)	1.169	0.28
Yes	7 (9.2)	18 (14.4)		
Renal tubular atrophy/interstitial fibrosis				
No	8 (10.5)	50 (40.0)	20.0	< 0.001
Yes	68 (89.5)	75 (60.0)		
Arteriolar hyalinosis				
No	42(55.3%)	84(67.2%)	2.897	0.09
Yes	34(44.7%)	41(32.8%)		
Arteriolosclerosis				
No	53(69.7%)	105(84.0%)	5.718	0.017
Yes	23(30.3%)	20(16.0%)		

### Analysis of risk factors for glomerular ischemic lesions

Based on the univariate analysis, the binary logistic regression analysis was conducted on the six variables (i.e., age, serum uric acid level, eGFR, serum albumin, TA/IF, and the presence of arteriolosclerosis) with significance in the univariate analysis. The results showed that the serum uric acid level and TA/IF were independent risk factors in GIL (*P* < 0.05) (Table 3).

**Table 3:** Binary Logistic regression analysis of risk factors of GIL.

Item	*B-*value	SD	Wald	Odds ratio	95% CI	*P*-value
Age	0.012	0.020	0.340	1.012	0.932 ~ 1.016	0.560
Serum uric acid level	0.012	0.003	20.490	1.012	1.007 ~ 1.017	< 0.001
eGFR	-0.027	0.022	1.555	0.973	0.932 ~ 1.016	0.212
Serum albumin	0.045	0.032	1.907	1.046	0.981 ~ 1.114	0.167
TA/IF	1.632	0.461	12.553	5.116	2.074 ~ 12.620	< 0.001
Arteriolosclerosis	0.322	0.414	0.602	1.379	0.612 ~ 3.108	0.438

### Serum uric acid level predicts GIL

**Figure 1:**
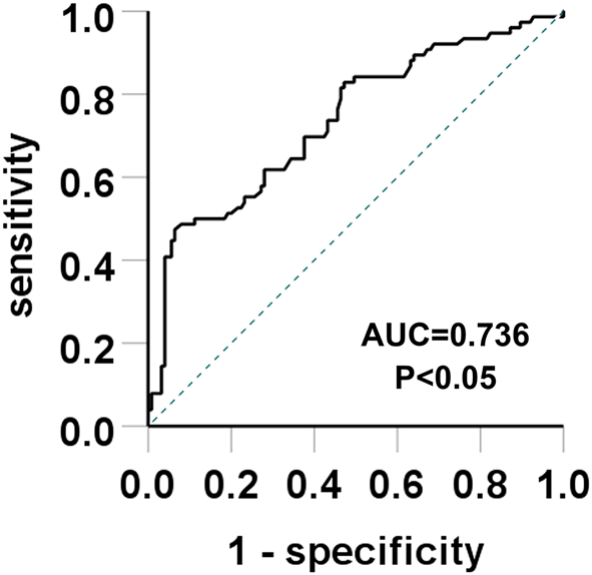
ROC curve highlighting the predictive effect of serum uric acid level on GIL in primary MN patients.

Finally, the effect of serum uric acid level on the GIL of PMN patients was determined by diagnostic ROC curves. The results showed that the AUC of serum uric acid level predicting GIL of PMN patients was 0.736 (*P* < 0.05) (Fig. 1). Its critical value and Youden’s index were 426.5 μmol/L and 0.41, respectively. Moreover, the sensitivity of predicting GIL was 47.4%, and the specificity was 93.6%, suggesting that serum uric acid levels greater than 426.5 μmol/L showed an essential value in predicting GIL.

## Discussion

Recently, the number of MN patients in China has significantly increased, with a younger onset age and some patients gradually progressing to end-stage kidney disease after only several years^2^. Exploring the risk factors of MN onset and timely intervention is crucial; however, there are limited studies on MN with GIL. Most research on ischemic renal diseases focuses on ischemic nephropathy caused by renal artery stenosis. The pathogenesis of ischemic renal diseases may be related to the decrease in renal blood perfusion exceeding the threshold of self-compensation, causing corresponding renal tubular damage, shrinkage of glomerular capillary loops, glomerulosclerosis, and gradual renal atrophy^8^. Our study found that the prevalence of GIL in MN was not low (37.8%), and there is increasing evidence that glomerular ischemic lesions play an important role in the progression of kidney disease^9–11^.

Hyperuricemia is a part of metabolic syndrome, and the prevalence shows an increasing trend. The prevalence of hyperuricemia in this study was 27.4%, slightly lower than that observed in another single-center study in the same province of China^12^. This difference might be related to recruiting PMN patients with normal renal function and excluding patients with secondary hyperuricemia. Epidemiology shows that not only is hyperuricemia an independent risk factor for cardiovascular and cerebrovascular events, but it is also a high-risk factor for the progression of chronic kidney disease (CKD)^13,14^. Previous studies have demonstrated that serum uric acid serves as an independent risk factor for the progression of IgAN, with a more pronounced impact observed among females compared to males^15,16^. Higher uric acid levels may also contribute to developing global and new renal damage in patients with lupus independently of other known risk factors^17,18^. Additionally, the latest research shows that hyperuricemia contributes to nephrosclerosis with ischemia and hyperfiltration^19^. Our study found that the prevalence of hypertension and LDL in the hyperuricemia group was significantly higher than those in the control group (*P* < 0.05), which was consistent with previous studies^20,21^. Additionally, we noted that the serum albumin in the hyperuricemia group was higher than that of the control group, which might be related to the richer diet of hyperuricemia patients on the premise that there was no significant difference between the two groups in urine protein quantitation. Hyperuricemia can lead to vasoconstriction and endothelial dysfunction; however, it remains unknown whether hyperuricemia causes GIL. In this study, the prevalence of GIL in the hyperuricemia group (69.1%) was significantly higher than in the control group (26.0%), suggesting that hyperuricemia might increase the risk of GIL. The prevalence of arteriosclerosis in the GIL was found to be significantly higher than that in the non-GIL according to our study findings. However, subsequent binary logistic regression analysis revealed that the arteriosclerosis prevalence did not emerge as an independent risk factor for glomerular ischemia. Although we rigorously selected PMN patients with normal renal function, the estimated glomerular filtration rate (eGFR) of the GIL group, as determined by univariate analysis, was significantly lower compared to that in the non-GIL group. However, subsequent multivariate logistic regression analysis revealed that the association between eGFR and GIL in this study was not statistically significant, suggesting that the decline in eGFR did not substantially impact the statistical outcomes of this investigation. The aforementioned findings may be attributed to the inclusion of patients with normal renal function (eGFR > 90ml/(min.1.73m^2^), thereby minimizing the impact of eGFR in this study.

The accumulation of uric acid in the kidney stimulates the degranulation of mast cells and the release of renin to promote the production of Ang II, leading to renal oxidative stress, mitochondrial structural damage, and microvascular system damage^22^. The presence of hyperuricemia is associated with the activation of both plasma renin and intra-kidney angiotensin activities, leading to renal vasoconstriction and potentially inducing ischemia in the kidney^23^. Furthermore, hyperuricemia causes oxidative stress and inflammation, which leads to the impairment of nitric oxide (NO) synthase (NOS) and consequent local vasoconstriction, ultimately leading to glomerular ischemia^24^. As mechanisms of CKD progression due to hyperuricemia, endothelial dysfunction induced by uric acid and activation of the Nod-like receptor protein 3 (NLRP3) inflammasome induced by urate crystals have been a focus of research^25,26^. Recently, ROS/NLRP3 inflammasome-mediated endothelial cell pyroptosis has been reported as the critical mechanism underlying the relationship between hyperuricemia and atherosclerosis^27^. Previous studies have shown that uric acid can induce the loss of glycocalyx in endothelial cells, which may be one of the mechanisms of endothelial dysfunction and kidney diseases^28^. The absence of the glycocalyx is the earliest sign of endothelial damage, leading to increased vascular permeability and a higher risk of intravascular thrombosis, resulting in microcirculation dysfunction, organ ischemia, and organ injury^29^. Recent studies have demonstrated a consistent association between hyperuricemia and renal damage, which is frequently attributed to the presence of insulin resistance (IR). In the context of hyperuricemia, elevated levels of uric acid significantly disrupt insulin signaling pathways, thereby diminishing insulin-induced eNOS activation and expression as well as NO synthesis in endothelial cells. Consequently, this cascade culminates in the development of endothelial cell IR, ultimately leading to impaired NO-dependent vasodilation and endothelial dysfunction that exacerbate renal ischemia^30^.

In the current study, we found that TA/IF was closely related to GIL. A conventional perspective on kidney diseases emphasizes the glomerular origin of disease progression, which initiates as glomerulosclerosis and subsequently induces hypoxia in the tubular segments. This leads to further tubular atrophy and interstitial fibrosis^31^. The latest evidence suggests that tubular interstitial damage may also contribute to an increased extent of glomerular damage^32^. Tubular interstitial fibrosis can induce tubule-to-glomerulus injury through the mechanism of tubuloglomerular crosstalk, thereby accelerating the progression of renal disease^33^. In human posttransplant kidneys, a follow-up of the development of glomerulosclerosis identified both interstitial fibrosis and tubular atrophy as independent predictors of subsequent glomerulosclerosis^34^. We conducted additional statistical analysis and categorized the study participants into two groups, namely the IF group and non-IF group, based on the results of kidney biopsy. Binary logistic regression analysis was performed on five variables that showed significant associations in univariate analysis: age, serum uric acid level, eGFR, serum albumin, and presence of arteriolosclerosis. The findings demonstrated that serum uric acid level remained an independent risk factor for GIL in the TA/IF group, while both serum uric acid level and eGFR were identified as independent risk factors for GIL in the non-TA/IF group. These results provide additional evidence supporting the role of uric acid in glomerular ischemia (refer to supplementary materials Tables S1-S2).

This study had several limitations. First, serum uric acid level was only measured once, without long-term follow-up data, resulting in some selective biases. Second, unintentional biases may have been introduced due to the cross-sectional retrospective design of this study in combination with a relatively small sample size. Multi-center, large sample, and RCT studies are required for further validation.

In summary, this study included 201 cases of PMN. The prevalence of GIL in the hyperuricemia group was higher than in the control group. Multivariate logistic regression analysis indicated that serum uric acid level and TA/IF off patients were independent risk factors for GIL. According to the ROC curves, the increase of uric acid showed predictive values in predicting GIL. When the serum uric acid level was greater than 426.5 μmol/L, the prevalence of GIL also increased. Therefore, clinicians and other medical professionals should focus on controlling serum uric acid levels in PMN patients. However, selecting the correct treatment to reduce GIL requires long-term follow-up and further research.

### Supplementary Information

Supplementary Table S1.

Supplementary Table S2.

## Data Availability

The data that support the findings of this study are available from the corresponding author upon reasonable request.
