# Supplementary material for: Serum uric acid level is associated with glomerular ischemic lesions in patients with primary membranous nephropathy: an analytical, cross-sectional study
Source: Sci Rep. 2024 Mar 29;14:7457. doi: 10.1038/s41598-024-57813-5 (PMC10978902; doi:10.1038/s41598-024-57813-5)
Supplement: Supplementary file 1 — Supplementary Table S1. [file 41598_2024_57813_MOESM1_ESM.doc]

Table S1 Binary Logistic regression analysis of risk factors for GIL in TA/IF group

Item	B-value	SD	Wald	P-value	Exp(B)	95%CI	
						Lower	Upper	
ALB	.027	.035	.588	.443	1.027	.959	1.100	
eGFR	-.003	.024	.011	.917	.997	.952	1.046	
Serum uric acid level	.010	.003	14.056	.000	1.010	1.005	1.016	
Age	.022	.022	.972	.324	1.022	.979	1.067	
Arteriolosclerosis(1)	.358	.433	.684	.408	1.431	.612	3.347	


         
