# Supplementary material for: Serum uric acid level is associated with glomerular ischemic lesions in patients with primary membranous nephropathy: an analytical, cross-sectional study
Source: Sci Rep. 2024 Mar 29;14:7457. doi: 10.1038/s41598-024-57813-5 (PMC10978902; doi:10.1038/s41598-024-57813-5)
Supplement: Supplementary file 2 — Supplementary Table S2. [file 41598_2024_57813_MOESM2_ESM.doc]

**Table S2** Binary Logistic regression analysis of risk factors for GIL in non-TA/IF group

| Item | B-value | SD | Wald | P-value | Exp(B) | 95%CI | |
| --- | --- | --- | --- | --- | --- | --- | --- |
|  |  |  |  |  |  | Lower | Upper |
| ALB | .192 | .114 | 2.850 | .091 | 1.212 | .970 | 1.515 |
| eGFR | -.148 | .064 | 5.319 | .021 | .862 | .760 | 0.978 |
| Serum uric acid level | .021 | .010 | 4.635 | .031 | 1.021 | 1.002 | 1.041 |
| Arteriolosclerosis(1) | -.111 | 2.206 | .003 | .960 | .895 | .012 | 67.499 |
| Age | -.031 | .050 | .388 | .534 | .970 | .879 | 1.069 |
